# Correlation between human development index and its components with COVID-19 indices: a global level ecologic study

**DOI:** 10.1186/s12889-022-13698-5

**Published:** 2022-08-15

**Authors:** Alireza Mirahmadizadeh, Mousa Ghelichi-Ghojogh, Mohebat Vali, Kimia Jokari, Haleh Ghaem, Abdolrasool Hemmati, Fatemeh Jafari, Seyed Sina Dehghani, Amir Hossein Hassani, Alireza Jafari, Fatemeh Rezaei

**Affiliations:** 1 Non-Communicable Diseases Research Center, School of Health, Shiraz University of Medical Sciences, Shiraz, Iran; 2 Student Research Committee, Shiraz University of Medical Sciences, Shiraz, Iran; 3 Non-Communicable Diseases Research Center, Department of Epidemiology, School of Health, Shiraz University of Medical Sciences, Shiraz, Iran; 4 Vice Chancellor Affairs, Shiraz University of Medical Sciences, Shiraz, Iran; 5 School of Medicine, Shiraz University of Medical Sciences, Shiraz, Iran; 6 Department of Health Education and Health Promotion, School of Health, Social Development and Health Promotion Research Center, Gonabad University of Medical Sciences, Gonabad, Iran; 7 Research Center for Social Determinants of Health, Jahrom University of Medical Sciences, Jahrom, Iran

**Keywords:** COVID-19, Coronavirus, Human development index, Ecologic study

## Abstract

**Background:**

Given that COVID-19 continues to spread worldwide, attempts to restrain the virus and to prevent the effects that critically ill patients with COVID-19 have on healthcare systems, has become a public health priority. This ecological study aimed to investigate the correlation between the Human Development Index (HDI) and the epidemiological indicators of COVID-19, including the cumulative incidence rate of cases, the cumulative incidence rate of death, performed COVID-19 tests per million, recovery rate, and case fatality rate.

**Methods:**

In this ecological study, a data set was provided, which included the epidemiologic indices of COVID-19, HDI, and its components for each country. Correlation coefficients were used to determine linear correlation. Also, the scatter plots of the HDI for the studied countries based on the epidemiologic indices of COVID-19 were drawn.

**Results:**

This study showed that HDI and its components had positive correlation with a cumulative incidence rate of cases, the cumulative incidence rate of death, and performed COVID-19 tests (*p* < 0.001). HDI and two of its components, including literacy and Gross National Income (GNI) components had negative correlation with case fatality rate (CFR). Also, HDI and two of its components, including literacy and life expectancy components had negative correlation with recovery rate.

**Conclusion:**

Our study showed that the HDI and its components can affect the epidemiological status of COVID-19. As HDI increased, the cumulative incidence rate of cases, cumulative incidence rate of death, and COVID-19 tests increased as well. As HDI increased, CFR and recovery rate decreased as well. Although the HDI is higher in high-income countries, these countries may have also better reporting and surveillance systems.

## Background

COVID-19 pandemic, caused by Severe Acute Respiratory Syndrome Coronavirus-2 (SARS-CoV-2), originated in Wuhan, China, in December 2019 and has spread worldwide [1, 2]. As of March 29th, 2020, COVID-19 has been reported by the World Health Organization (WHO) in more than 100 countries (most prevalent in the United States, Italy, China, Spain, Germany, Iran, and France) [3] and has also been named as a health emergency. So, it was introduced to the public and international attention [4]. According to WHO, the total number of definite cases of the disease as of December 19th, 2020, was 73,996,237, and the number of deaths was 1,663,474. The total number of cases was 31,925,704 in the Americas, 23,168,215 in Europe, 11,572,247 in Southeast Asia, 4,641,968 in the eastern Mediterranean, 1,687,168 in Africa, and 999,891 in the western Pacific [5]. The Human Development Index (HDI) measures the average achievement of a country based on three dimensions: 1. Longevity, which indicates the ability of individuals to enjoy a long and healthy life. 2. The level of access of the population to knowledge which is measured through the educational system. 3. The average life standards of people which is measured based on purchasing power [6]. Past research has shown that public health outcomes are affected by HDI indices such as education, employment, and income, probably because these variables affect access to public health infrastructure and health facilities [7]. Some researchers believe that the situation of COVID-19 epidemic worldwide can be projected considering some macro variables such as population size, HDI, and migration rate [8]. Deaths from infectious diseases and respiratory infections are higher in countries with lower HDIs [9]. Still, a study in Italy found that HDI was directly related to infection rates and COVID-19 mortality rates [10]. This study covers only one country and only two factors: infection rates and COVID-19 mortality rates. As regards, COVID-19 has created a significant burden in the world [11]. Assessing the incidence and mortality of the disease and its associated risk factors may help us to better understand the nature and course of this disease [7]. Moreover, although the etiology of COVID-19 has been extensively studied, many aspects of the disease are still unknown and its socioeconomic elements have been less studied. Also, most previous studies have only looked at the changes in HDI components after COVID-19, not the effect of HDI components on disease [12–14]. HDI is also one of the factors affecting the incidence and mortality of patients [15], but there is little evidence about the role of social development in the control of COVID-19. Previous studies have not addressed the role of HDI and its components in cumulative cases, cumulative deaths, case fatality rate, recovery rate, and the number of performed COVID-19 tests globally. Therefore, the present study aimed to investigate the correlation between HDI and epidemiological indices of coronavirus disease, including the cumulative incidence rate of cases, the cumulative incidence rate of death, performed COVID-19 tests, recovery rate, and case fatality rate.

## Methods

### Study design and data collection

This survey is an ecological study, so all studied variables are aggregate variables. To collect the variables in the study, a data set was provided, which included the information of each country based on the cumulative cases, cumulative deaths, case fatality rate, recovery rate, the number of performed COVID-19 tests, HDI, and its components. Information about COVID-19 for each country was retrieved from https://www.worldometers.info/ for the period from the date of the first reported case until November 30th, 2020. In this study, the relationship between COVID-19 indices, including cumulative incidence rate of cases, cumulative incidence rate of death, cumulative tests performed, recovery rate, case fatality rate, and HDI, were investigated. Data on HDI and its components, including literacy, life expectancy, and Gross National Income (GNI) in 2019, were also collected from human development reports [16]. The HDI is defined as follows: A measure of development using an index which includes three main sections (called dimensions) with separate indicators feeding into three separate indices, which are equally weighted in the final HDI: life expectancy index, Knowledge (education index) and GNI per capita [17]. Life expectancy index is the average number of years that a newborn could expect to live if they were to pass through life exposed to the sex- and age-specific death rates prevailing at the time of their birth, for a specific year, in a given country, territory, or geographic area [18]. Also, the education index is measured by the adult literacy rate (with two-thirds weighting) and the combined primary, secondary, and tertiary, gross enrollment ratio (with one-third weighting). The adult literacy rate gives an indication of the ability to read and write [19]. The GNI measures the extent to which the distribution of income (or in some cases, consumption expenditure) among individuals or households within an economy deviates from a perfectly equal distribution. A GNI of 0 represents perfect equality, while an index of 100 implies perfect inequality [20]. HDI data and its components were available for 157 countries. The data regarding cumulative incidence rate of cases, cumulative incidence rate of death, cumulative tests performed, recovery rate, and case fatality rate were available for 157, 151, 146, 152, and 153 countries respectively.

### Statistical analysis

Scatter plots of HDI for the studied countries were drawn based on cumulative incidence rate of cases, cumulative incidence rate of death, tests performed, recovery rate, and case fatality rate of COVID-19. Furthermore, Spearman correlation coefficient was also used to verify the correlation between HDI and indicators related to COVID-19. The correlation coefficient was categorized as very weak (*r* = 0.00– < 0.20), week (*r* = 0.20– < 0.40), moderate (*r* = 0.40– < 0.60), strong (*r* = 0.60– < 0.70), and very week (*r* = 0.80–1.00) [21]. Also, R-squared (R^2^) was calculate. The proportion of variance in the dependent variable which is described by the independent variables in the sample depicts by R^2^ [22]. Which always lies between 0 and 1. Statistical analyses were performed with Statistical Package for Social Science (IBM SPSS Statistics for Windows, Version 26.0. Armonk, NY: IBM Corp). *P*-values less than 0.05 were considered as statistically significant.

## Results

This ecological study showed that among all countries surveyed, Montenegro (60,310.56 per million) had the highest cumulative incidence rate of COVID-19, while Tanzania (8.42 per million) had the lowest cumulative incidence rate of COVID-19. Also, it revealed that Belgium (1425.15 per million) had the highest cumulative incidence rate of death due to COVID-19 when Burundi (0.08 per million) had the lowest cumulative incidence rate of death due to COVID-19. Luxembourg (2,180,641.18 per million) was the country with the highest number of performed COVID-19 tests per million among studied countries. The lowest number of COVID-19 tests per million belonged to Yemen (560.05 per million). The highest recovery rate was in Timor-Leste (100%) when the lowest rate was in Belgium (6.48%). In addition, the highest case fatality rate of COVID-19 was in Yemen (28.34%) while the lowest case fatality rate of COVID-19 was in Singapore (0.05%). Also, the highest HDI was in Norway and Switzerland (0.95), and the lowest in Niger (0.38). At birth, the highest life expectancy was in Japan (84.63 years) and the lowest in Chad (54.24 years). The highest mean years of schooling were in Germany (14.15 years) and the lowest in Burkina Faso (1.64 years). The highest GNI was in Qatar (92,418.2 dollars per capita) when the lowest was in Burundi (753.9 dollars per capita). Table 1 shows the correlation coefficient between the indicators related to COVID-19 and the HDI. This study showed that HDI and its components had positive correlation with the cumulative incidence rate of cases, the cumulative incidence rate of death, and performed COVID-19 tests (*p* < 0.001). As HDI and three components increases, the cumulative incidence rate of cases, the cumulative incidence rate of death, and performed COVID-19 tests, will increase. Also, the case fatality rate had negative correlation with HDI and two of its components, including literacy and GNI (*P* < 0.05). With increasing HDI, literacy, and GNI, the case fatality rate will decrease. The correlation between HDI and two of its components, including literacy and life expectancy with the recovery rate was also negative; with increased HDI, literacy, and life expectancy, recovery rate decreased (*P* < 0.05). In countries with a population of 10 million and more, a significant positive correlation was observed between HDI and its components with the cumulative incidence rate of cases, the cumulative incidence rate of death, and performed COVID-19 tests (*p* < 0.001). However, HDI and its dimensions were not correlated with recovery rate and case fatality rate.

**Table 1:** Spearman correlation of indices related to COVID-19 with HDI and its components

Variable	All countries					Countries with ≥ 10 million population				
	N	Correlation coefficient	R^2^	*p*-value	Comment	N	Correlation coefficient	R^2^	*p*-value	Comment
Cumulative Incidence rate of Cases (per million)										
HDI	157	0.67	0.35	< 0.001	strong	85	0.75	0.44	< 0.001	strong
Mean years of schooling	157	0.63	0.30	< 0.001	strong	85	0.69	0.36	< 0.001	strong
Life expectancy	157	0.65	0.33	< 0.001	strong	85	0.67	0.36	< 0.001	strong
GNI	157	0.68	0.30	< 0.001	strong	85	0.75	0.44	< 0.001	strong
Cumulative Incidence rate of Death (per million)										
HDI	152	0.55	0.24	< 0.001	moderate	82	0.68	0.37	< 0.001	strong
Mean years of schooling	152	0.51	0.19	< 0.001	moderate	82	0.59	0.27	< 0.001	moderate
Life expectancy	152	0.55	0.24	< 0.001	strong	82	0.64	0.34	< 0.001	strong
GNI	152	0.53	0.10	< 0.001	moderate	82	0.69	0.34	< 0.001	strong
Tests performed per million										
HDI	146	0.85	0.30	< 0.001	very strong	78	0.86	0.53	< 0.001	very strong
Mean years of schooling	146	0.76	0.23	< 0.001	strong	78	0.79	0.46	< 0.001	strong
Life expectancy	146	0.79	0.25	< 0.001	strong	78	0.80	0.43	< 0.001	very strong
GNI	146	0.86	0.49	< 0.001	very strong	78	0.86	0.66	< 0.001	very strong
Case Fatality Rate (%)										
HDI	153	-0.20	0.05	0.01	weak	84	0.02	0.02	0.86	NS^a^
Mean years of schooling	153	-0.23	0.06	0.004	weak	84	-0.07	0.04	0.54	NS
Life expectancy	153	-0.13	0.02	0.11	NS	84	0.10	0.00	0.36	NS
GNI	153	-0.23	0.04	0.004	weak	84	0.06	0.00	0.59	NS
Recovery rate (%)										
HDI	152	-0.18	0.05	0.03	very weak	80	-0.15	0.05	0.19	NS
Mean years of schooling	152	-0.21	0.06	0.01	weak	80	-0.11	0.03	0.31	NS
Life expectancy	152	-0.20	0.05	0.01	weak	80	-0.20	0.07	0.07	NS
GNI	152	-0.13	0.02	0.11	NS	80	-0.17	0.09	0.12	NS

^a^Indicates not significant

**Fig. 1:**
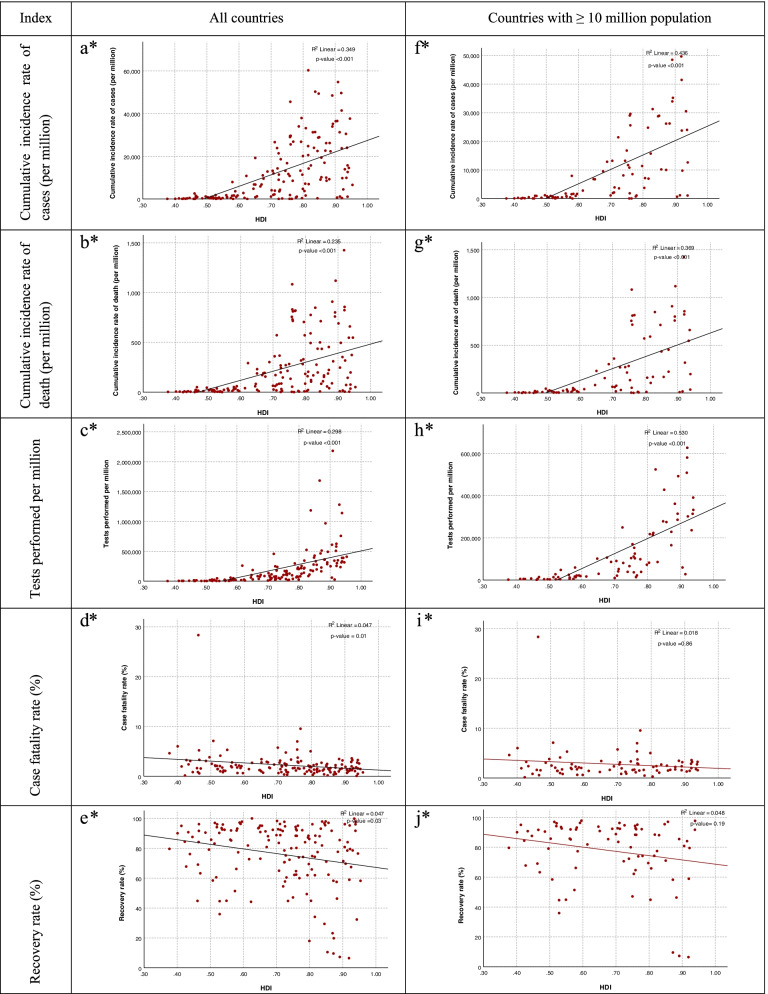
Scatter plot of correlation between HDI with indices related to COVID-19. ^*^a: Norway, Switzerland, Ireland, Germany, Iceland, Australia, Sweden, Singapore, the Netherlands, Denmark, Finland, Canada, New Zealand, United Kingdom, United States, Belgium, Japan, Austria, Luxembourg, Israel, Republic of Korea, Slovenia, Spain, France, Czech Republic, Malta, Italy, Estonia, Cyprus, Greece, Poland, Lithuania, United Arab Emirates, Saudi Arabia, Slovak Republic, Latvia, Portugal, Qatar, Chile, Hungary, Brunei Darussalam, Bahrain, Croatia, Oman, Argentina, Russia, Kazakhstan, Belarus, Montenegro, Bulgaria, Romania, Kuwait, Uruguay, Turkey, Malaysia, Serbia, Trinidad and Tobago, Iran, Mauritius, Panama, Costa Rica, Albania, Georgia, Sri Lanka, Cuba, Bosnia and Herzegovina, Mexico, Thailand, Brazil, Colombia, Armenia, North Macedonia, Peru, Algeria, Ecuador, China, Azerbaijan, Ukraine, Dominican Republic, Tunisia, Mongolia, Lebanon, Botswana, Venezuela RB, Jamaica, Paraguay, Fiji, Suriname, Jordan, Belize, Maldives, Philippines, Moldova, Uzbekistan, Libya, Indonesia, South Africa, Gabon, Egypt, Iraq, Morocco, Kyrgyz Republic, Guyana, El Salvador, Tajikistan, Nicaragua, Guatemala, Cabo Verde, India, Namibia, Timor-Leste, Honduras, Bhutan, Bangladesh, Ghana, Zambia, Equatorial, Guinea, Myanmar, Cambodia, Nepal, Kenya, Angola, Zimbabwe, Cameroon, Pakistan, Syrian Arab Republic, Papua New Guinea, Comoros, Rwanda, Nigeria, Tanzania, Uganda, Mauritania, Madagascar, Benin, Lesotho, Senegal, Togo, Sudan, Haiti, Afghanistan, Malawi, Ethiopia, the Gambia, Guinea, Liberia, Yemen Republic, Guinea-Bissau, Democratic Republic of Congo, Mozambique, Sierra Leone, Burkina Faso, Mali, Burundi, South Sudan, Chad, and Niger. ^*^b: all countries in (a) except Mongolia, Timor-Leste, Bhutan, Cambodia, and Tanzania. ^*^c: all countries in (a) except Sudan, Chad, Syrian Arab Republic, Tanzania, Sierra Leone, Algeria, Nicaragua, Burkina Faso, Democratic Republic of Congo, Comoros, and Tajikistan. ^*^d: all countries in (a) except Timor-Leste, Cambodia, Bhutan, and Mongolia. ^*^e: all countries in (a) except Spain, United Kingdom, Sweden, the Netherlands, and Democratic Republic of Congo. ^*^f: all countries in (a) except Countries with < 10 million population. Countries with < 10 million population include Albania, Armenia, Austria, Bahrain, Belarus, Belize, Bhutan, Bosnia and Herzegovina, Botswana, Brunei Darussalam, Bulgaria, Cabo Verde, Comoros, Costa Rica, Croatia, Cyprus, Denmark, El Salvador, Equatorial Guinea, Estonia, Fiji, Finland, Gabon, Gambia, The, Georgia, Guinea-Bissau, Guyana, Honduras, Hungary, Iceland, Ireland, Israel, Jamaica, Kuwait, Kyrgyz Republic, Latvia, Lebanon, Lesotho, Liberia, Libya, Lithuania, Luxembourg, Maldives, Malta, Mauritania, Mauritius, Moldova, Mongolia, Montenegro, Namibia, New Zealand, Nicaragua, North Macedonia, Norway, Oman, Panama, Papua New Guinea, Paraguay, Qatar, Serbia, Sierra Leone, Singapore, Slovak Republic, Slovenia, Suriname, Switzerland, Tajikistan, Timor-Leste, Togo, Trinidad and Tobago, United Arab Emirates, and Uruguay.^*^g: all countries in (a) except Mongolia, Timor-Leste, Bhutan, Cambodia, Tanzania, and Countries with < 10 million population. ^*^h: all countries in (a) except Sudan, Chad, Syrian Arab Republic, Tanzania, Sierra Leone, Algeria, Nicaragua, Burkina Faso, Democratic Republic of Congo, Comoros, Tajikistan, and Countries with < 10 million population. ^*^ **i**: all countries in (a) except Timor-Leste, Cambodia, Bhutan, Mongolia and Countries with < 10 million population. ^*^ **j**: all countries in (a) except Spain, United Kingdom, Sweden, Netherlands, Congo. Dem. Rep

Figure 1, depicts the scatter plot of HDI by cumulative incidence rate of cases, cumulative incidence rate of death, performed COVID-19 tests, recovery rate, and case fatality rate in all countries and the countries with a population of 10 million and more. The strength of the linear relationship between two quantitative variables shows by scatter plots. In the section of all countries, the highest R^2^ was observed in the cumulative incidence rate of cases (R^2^ = 0.349) and the performed COVID-19 tests (R^2^ = 0.298). HDI explain about 35% and 30% of the variance in the cumulative incidence rate of cases and the performed COVID-19 tests, respectively. In countries with a population of 10 million or more, the highest R^2^ was for the tests performed (R^2^ = 0.530), followed by the cumulative incidence rate of cases (R^2^ = 0.436). In countries with a population of 10 million or more HDI explain about 53% and 44% of the variance in the performed COVID-19 tests and cumulative incidence rate of cases, respectively.

## Discussion

COVID-19 is a global health crisis in our time and the biggest challenge that the world is encountering [23]. In this pandemic, a critical evaluation of epidemiological data is needed to understand the viral transmission dynamics at the local, regional, and global scales. The continuous integration of this data stream with access to resources and the development of countries can be helpful to reduce COVID-19 transmission [24]. There has been a lack of research about how macroeconomic determinants relate to the epidemiological status of COVID-19; the evaluation of the relationship between COVID-19 epidemiological indicators and specific components of HDI (education, health, and living standards) shows their relative importance in this pandemic. HDI is an index that measures key dimensions of human development. The three key dimensions are: 1- Long and healthy life (Measured by life expectancy) 2-Access to education (is measured by the expected years of education of children at school age and the average years of education in the adult population) 3-and a decent standard of living (which is measured by the per capita gross national income adjusted for the price level of the country)[25].

Our study showed that the higher the HDI, the higher the cumulative incidence rate of the cases, the cumulative incidence rate of death, and performed tests. This correlation may be due to the strong infrastructure in these countries, these countries with higher HDI have been able to record more laboratory tests due to higher capacity. The high incidence in nations with a higher HDI may also be linked to the strength of an excellent health care system for early identification and detection of asymptomatic and subclinical kinds of illness, as well as more effective screening program implementation in these countries. Poor access to diagnostic equipment decreases the frequency of infection in nations with a low HDI. Furthermore, these nations' illness reporting systems are of poor quality, resulting in low disease reporting [26]. It has been confirmed in previous studies that countries with lower data quality are mostly countries with lower levels of development [27]. Therefore, due to the fact that a pandemic can be extinguished when there is better detection in that area, this may be a reason that countries with higher HDI could do a better performance against COVID-19. Also, about HDI components, there was a negative correlation between literacy and GNI with case fatality rate. However, the previous study states, showed that with increasing mean of school years, the incidence and mortality rate for COVID-19 increased. And is expressed, The incidence increases with increasing health literacy and awareness of the early symptoms of the disease and early diagnosis [26]. Also, in the all countries, the highest R^2^ was observed in the cumulative incidence rate of cases and the performed COVID-19 tests. HDI explain about 35%, 30%, and 24% of the variance in the cumulative incidence rate of cases, the performed COVID-19 tests, and cumulative incidence rate of death, respectively. In countries with a population of 10 million or more HDI explain about 53%, 44%, and 37% of the variance in the performed COVID-19 tests, cumulative incidence rate of cases, and cumulative incidence rate of death, respectively. Therefore, in addition to the HDI, other factors may be related to these indices.

Also, there was a negative correlation between literacy and life expectancy with recovery rate in our study. Life expectancy at birth is one of the components of HDI. A rise in the old population will occur as life expectancy increases. According to prior research, the elderly has the greatest prevalence and mortality of Covid-19. When it comes to the link between age and the occurrence and mortality of this illness, it can be noted that older individuals are more susceptible to severe instances of the disease since their immune systems are weaker against the disease [28–30]. This negative relationship may be the result of the fact that with increasing life expectancy in a society, the society becomes more prone to ageing, and due to the more difficult disease conditions for the elderly, the recovery rate in this type of population decreases. Therefore, it can be said that the extent of damage caused by the epidemic depends on the region in which people live and the social and economic conditions under which they spend their lives [31]. Previous studies have shown that, the elderly is the most at risk in many developed countries, the high COVID-19 mortality rate has been attributed to a higher rate than the elderly. Patients over 50, particularly those aged 60 to 69, are at the greatest risk of dying from Covid-19. The smaller proportions of the older age group are partially responsible for low Covid-19 mortality per million population and high Covid-19 mortality in adults in nations with high birth rates. Covid 19 is less likely to infect countries with a large youth population. This may boost herd immunity and function as a barrier to disease transmission, delaying or stopping the illness from spreading to others [32]. However, a prior research found that socioeconomic vulnerability patterns impact Covid-19 prevalence and mortality more than population age structure or the prevalence of existing chronic conditions [33].

So, in general, the positive spatial correlations between the incidence of COVID-19 and the human development measured by HDI are unprecedented. Generally, epidemiological studies of infectious diseases have found an indirect indicator of health in HDI because the highest value of this index is related to life expectancy, income, and education [34]. This set of factors can confirm the results of our study, showing that high HDI can facilitate severe viral circulation, transmission, and clinical regeneration of COVID-19. Low levels of HDI depicts not only population vulnerabilities but also health care problems in diagnosis and treatment of the disease and generally indicates the fragility of health services, especially in dealing with epidemics [35]. Even in wealthy countries, such as the United States, social inequalities in cities determine the high or low risk of disease for their residents. For example, in Boston, increased poverty and the prevalence of diseases in certain areas as well as good living conditions, and low prevalence of these diseases in other places proves this fact [36, 37]. Another study in Italy found that HDI was directly related to infection rate and COVID-19 mortality rate [10].This scenario asserts the relationship between social policies and the health conditions experienced by people. Khalatbari-Soltani et al. [38]. Emphasized on the need to supplement the WHO standard reports on COVID-19 by measuring socioeconomic position (SEP). They discussed that the WHO clinical report focused on age, sex, places of diagnosis, and residence. At the same time, other elements in the social environment such as occupation, income, or education may be predictive of the disease.

Ecological studies have a history of use in public health issues and diseases [39, 40]. In this study, limitations related to the method used must be considered and the results must be carefully interpreted as there may be an ecological fallacy. In ecological studies, observing the relationship between two variables at the collective level does not necessarily mean that this relationship remains the same in the individual level. However, this study had a good internal validity because the data represent the geographical strata analyzed and examined for more than 150 countries. However, it is essential to be more careful in interpreting cases since the low efficacy of reporting systems, which can be affected by inequal access to diagnostic tests and variation in healthcare quality.

### Study strengths and limitations

One of the strengths of the present study is that for the first time, the relationship between HDI and COVID-19 epidemiological indices including, mortality rate, recovery, especially new cases, and the number of tests, was examined in 157 countries. But the limitation of this study ecologic fallacy and using data at the aggregate level due to its nature.

## Conclusion

Our study showed that the HDI and its components can affect the epidemiological status of COVID-19 disease. As HDI increased, the cumulative incidence rate of cases, cumulative incidence rate of death, and COVID-19 tests increased as well. As HDI increased, CFR and recovery rate decreased as well. Although the HDI is higher in high-income countries, these countries may have also better reporting and surveillance systems. It seems that global assistance to countries with less HDI can effectively reduce the pandemic crisis in these countries and other regions. As in respiratory epidemics, borders cannot be a factor in disease prevention, if international cooperation is not practiced, we will not move towards disease control.

## Data Availability

The datasets generated during and analyzed during the current study are not publicly available due to privacy of the study project but are available from the corresponding author on reasonable request.

## Abbreviations

HDI: Human Development Index
WHO: World Health Organization
GNI: Gross National Income
